# Detection of a strongly negative surface potential at Saturn's moon Hyperion

**DOI:** 10.1002/2014GL061127

**Published:** 2014-10-16

**Authors:** T A Nordheim, G H Jones, E Roussos, J S Leisner, A J Coates, W S Kurth, K K Khurana, N Krupp, M K Dougherty, J H Waite

**Affiliations:** 1 Mullard Space Science Laboratory, University College LondonDorking, UK; 2 Centre for Planetary Sciences at UCL/BirkbeckLondon, UK; 3 Max Planck Institute for Solar System ResearchGöttingen, Germany; 4 Institute for Theoretical Physics, Braunschweig University of TechnologyBraunschweig, Germany; 5 Now at SDSE, LLCSilver Spring, Maryland, USA; 6 Department of Physics and Astronomy, University of IowaIowa City, Iowa, USA; 7 Institute of Geophysics and Planetary Physics, University of CaliforniaLos Angeles, California, USA; 8 Blackett Laboratory, Imperial College LondonLondon, UK; 9 Southwest Research InstituteSan Antonio, Texas, USA

**Keywords:** Saturn's moons, surface charging, Cassini, Hyperion

## Abstract

On 26 September 2005, Cassini conducted its only close targeted flyby of Saturn's small, irregularly shaped moon Hyperion. Approximately 6 min before the closest approach, the electron spectrometer (ELS), part of the Cassini Plasma Spectrometer (CAPS) detected a field-aligned electron population originating from the direction of the moon's surface. Plasma wave activity detected by the Radio and Plasma Wave instrument suggests electron beam activity. A dropout in energetic electrons was observed by both CAPS-ELS and the Magnetospheric Imaging Instrument Low-Energy Magnetospheric Measurement System, indicating that the moon and the spacecraft were magnetically connected when the field-aligned electron population was observed. We show that this constitutes a remote detection of a strongly negative (∼ −200 V) surface potential on Hyperion, consistent with the predicted surface potential in regions near the solar terminator.

## Key Points

- Cassini detected evidence of surface charging on Saturn's moon Hyperion
- Strong negative surface potential caused electron beam between moon and Cassini
- Observed potential compares well with predicted potential near terminator

## 1 Introduction

Hyperion is a highly irregular outer moon of Saturn, with dimensions of 180 × 133 × 103 km and a mean radius of 133 ± 8 km. It has a low mean density, indicating that it may consist primarily of water ice with an unusually high degree of porosity (>40%), resulting in its characteristic “sponge-like” appearance [*Thomas et al*., 2007]. Observations by the Voyager and Cassini spacecraft found that Hyperion has a chaotic spin state, rotating nearly about its long axis by 72–75°/d [*Thomas*, 1995; *Thomas et al*., 2007]. Its orbital semimajor axis is 24.55 Saturn radii (1 *R_s_* = 60,268 km), which takes it outside Saturn's magnetosphere for parts of its orbit. Hence, Hyperion is exposed to plasma conditions representative of the outer magnetosphere, magnetosheath, or solar wind, depending on its orbital position and the overall magnetospheric configuration at the time.

Due to interaction with solar photons and magnetospheric plasma, it is expected that surfaces in Saturn's magnetosphere may acquire a net electric charge. Impinging solar UV photons will tend to drive these surfaces toward positive potentials through photoelectron emission, whereas impinging plasma will tend to drive them toward negative potentials due to the thermal electron flux [*Whipple*, 1981]. Incident electrons may also liberate secondary electrons from the surface, with a yield that is related to the kinetic energy of incident electrons [*Sternglass*, 1954]. Thus, for a given surface, the potential is given by the balance of the currents

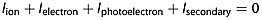


Previous theoretical studies have considered surface charging at several of Saturn's large moons [*Roussos et al*., 2010], its small moon Atlas [*Hirata and Miyamoto*, 2012] as well as *E* ring grains [*Horanyi et al*., 1992; *Jurac et al*., 1995; *Kempf et al*., 2006]. In particular, *Roussos et al*. [2010] considered the trailing (plasma-absorbing) hemispheres of Mimas, Rhea, Dione, and Tethys, with predicted surface potentials ranging from a few volts positive to more than 150 V negative, depending on the local solar zenith and plasma flow angles.

As Hyperion is expected to be an unmagnetized object that does not contribute to significant mass loading of the Saturnian magnetic field, we would expect that its magnetospheric interaction is that of a simple plasma absorber, with a dropout in low-energy plasma inside the moon's wake and a dropout (microsignature) in energetic electrons and ions for some distance from the moon at its L shell.

We report on the discovery of a strongly negative surface potential at Hyperion by the Cassini low-energy Electron Spectrometer (ELS), part of the Cassini Plasma Spectrometer (CAPS) [*Young et al*., 2004]. CAPS-ELS detects the energy per charge ratio of negative particles from 0.6 eV/e to 28.8 keV/e with an energy resolution (Δ*E*/*E*) of 16.7%. The instrument consists of eight anodes that are each 20° × 5° across and oriented in a 160° fan. Also presented are measurements made by the Magnetospheric Imaging Instrument Low-Energy Magnetospheric Measurement System (MIMI-LEMMS) [*Krimigis et al*., 2004], the Radio and Plasma Wave (RPWS) instrument [*Gurnett et al*., 2004], and magnetometer (MAG) [*Dougherty et al*., 2004], which provide crucial context for the CAPS-ELS observations.

## 2 Hyperion Encounter

The only close flyby of Hyperion occurred on 26 September 2005 (day of year 269), when the Cassini spacecraft passed within 520 km (∼3.9 mean Hyperion Radii) of the nominal moon surface. During this time, Hyperion was at 05:15 Saturn local time (LT), and the spacecraft passed just south of the moon's plasma wake if nominal corotation is assumed. Around the time of the encounter, Cassini was inside Saturn's magnetosphere and close to the current sheet, as evidenced by frequent reversals of the radial component of the magnetic field during most of the day. It is therefore likely that both Cassini and Hyperion were in the Saturnian plasma sheet when the flyby occurred. The Cassini magnetometer (MAG) [*Dougherty et al*., 2004] did not observe any clear magnetic field signature that could be associated with Hyperion, which is consistent with a simple plasma absorber, and the fact that the spacecraft did not pass through the moon's plasma wake.

**Figure 1:**
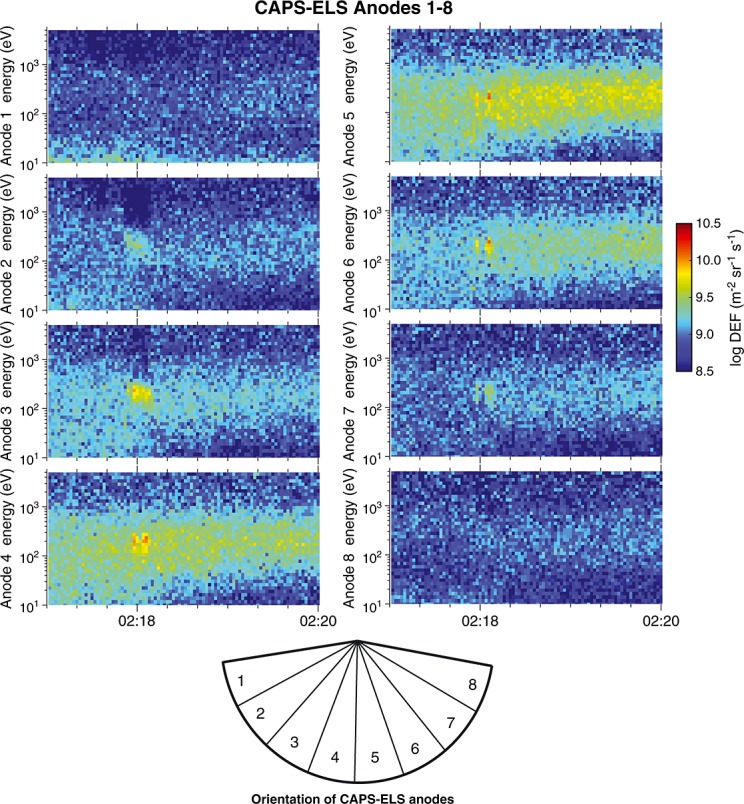
CAPS-ELS spectrograms for anodes 1–8 near 02:18 UTC when the electron feature is detected. The feature is observed as an increase in differential energy flux at 160–280 eV of more than an order of magnitude above the background level.

**Figure 2:**
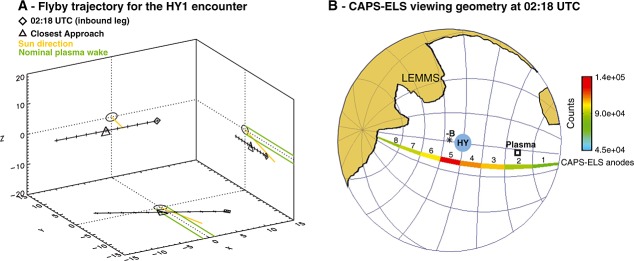
(a) The trajectory of the Cassini spacecraft during the HY1 flyby. The Sun direction (yellow) and nominal plasma wake (green) are indicated. Units are in Hyperion radii. (b) CAPS-ELS viewing geometry during the 26 September 2005 Hyperion flyby. This figure shows the instrument's hemispheric field of view from 02:18:09 to 02:18:11 UTC with the instantaneous anode look directions colored by counts in the 274 eV bin. Hyperion's disk, the observed magnetic field (−*B*) and the nominal arrival direction of corotating thermal plasma are shown for reference. The shaded regions show where portions of Cassini and its instruments obstruct CAPS viewing.

At roughly 02:18 UTC, approximately 6 min (∼2233 km, 16.79 Hyperion radii) before the closest approach, the CAPS-ELS instrument observed more than an order-of-magnitude increase in the 160–280 eV differential energy flux in two sharp peaks (Figure 1). The electron enhancement was directional, being most prominent in the CAPS-ELS anodes 4, 5, and 6: those pointed toward the disk of Hyperion and along the magnetic field (Figure 2). The pitch angle distribution of these electrons was confined to < 35°, indicating that this was a roughly field-aligned population.

**Figure 3:**
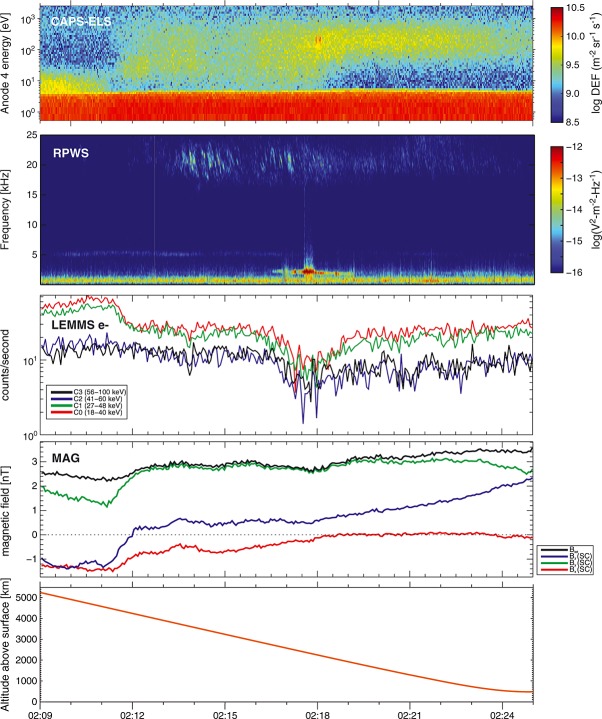
Summary plot showing data from CAPS-ELS anode 4, LEMMS channels C0–C4, RPWS, and MAG during the time of the Hyperion encounter. The low-energy electron feature is observed at 160–280 eV in CAPS-ELS concurrently with an intense 2 kHz plasma wave feature observed by RPWS and a dropout in 18–100 keV electrons observed by MIMI-LEMMS.

**Figure 4:**
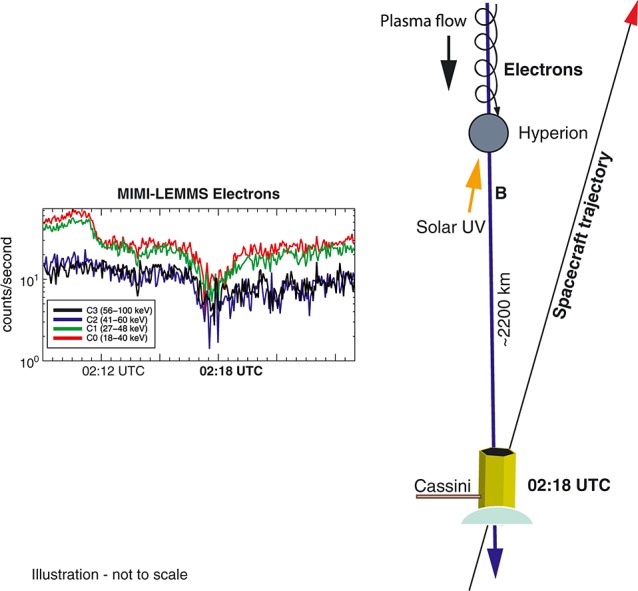
Illustration showing how a magnetic connection between the spacecraft and Hyperion lead can lead to the reduction in 18–100 keV electron counts observed by LEMMS. Not to scale.

At this time, the Saturnian magnetic field sampled by Cassini appeared to pass close to the moon's surface, assuming that the magnetic field direction remained linear over the distance between the spacecraft and Hyperion. However, as the Saturnian magnetic field is highly variable in this region of the magnetosphere, determining an exact surface location for the magnetic footprint is not possible. Concurrently, a foreground electron flux depletion in the 500–5000 eV range was seen in CAPS-ELS anodes 2 and 3, which observed electrons with pitch angles of 40–70°, indicating the low-energy end of an energetic particle dropout due to Hyperion. A broad depletion in high-energy electrons was also seen in the C0–C3 channels of the MIMI-LEMMS instrument (Figure 3), corresponding to a dropout in number densities of electrons with energies of 18–100 keV, which is consistent with a region partially evacuated of energetic particles due to absorption by Hyperion (Figure 4). The gyroradii of 500 eV electrons at pitch angles of 40–70° and a *B* of 2.69 nT are 18.1–26.3 km, respectively. Based on these observations, we conclude that it is easily plausible that the Cassini spacecraft was magnetically connected to the surface of Hyperion when the electron feature in CAPS-ELS was observed and that the observed field-aligned electron population is Hyperionian in origin.

When the CAPS-ELS feature was observed, the RPWS instrument observed an intense plasma wave feature near 2 kHz (Figure 3), with no magnetic component above the instrument threshold. This is consistent with the signature of a Langmuir wave, which may occur due to plasma density perturbations in the presence of electron beams [*Gurnett and Bhattacharjee*, 2005].The frequency at which the feature appeared is consistent with the plasma frequency for plasma densities of 0.0035–0.0055 cm^−3^, which compares well with CAPS measurements of the local plasma density during the encounter (Table 1). The observed feature is similar to the electrostatic wave activity reported by *Santolík et al*. [2011] during Cassini's 2 March 2010 flyby of Rhea. That feature occurred near the local electron plasma frequency and was attributed to a low-energy (∼35 eV) electron beam accelerated up from the surface of the moon simultaneously observed by CAPS-ELS during the flyby.

**Table 1:** Model Input Parameters for Hyperion Flyby

Parameter	Value	Note
*T_e_*	60 eV	Cassini CAPS measurements
*N_e_*	0.05 cm^−3^	Cassini CAPS measurements
*V_r_*	150 km s^−1^	Estimated
*T_i_*	500 eV	Estimated
Ion mass	12 amu	Estimated
*E*_max_	340 eV	*Jurac et al*. [1995]
*δ*_max_	2.35	*Jurac et al*. [1995]
*I_p_*	6.11E^−08^ A m^−2^	Scaled from *Sternovsky et al*. [2008]
*B*_tot_	2.69 nT	Cassini MAG measurements

During the time of the Hyperion encounter, the Cassini spacecraft was charged to a slightly positive potential of ∼5 V, which can be inferred from the presence of spacecraft photoelectrons at low energies in the CAPS-ELS measurements as seen in Figure 3. Ion data from the CAPS Ion Beam Spectrometer and Ion Mass Spectrometer were examined, but showed no signs of moon-related signatures during the encounter.

## 3 Surface Charging Calculations

The expected surface potential of Hyperion during the flyby was evaluated using the model of *Roussos et al*. [2010], which is based on the formulation of *Manka* [1973] for Earth's Moon and modified for the decoupling between solar illumination and plasma flow angles onto the surfaces of the Saturnian moons. During the encounter, Hyperion was near dawn (0600 LT), which means that the direction of solar UV flux was nearly 180° to the direction of the nominal corotation plasma flow.

The secondary electron yield (*δ*) is determined according to the angle-averaged form of the Katz formula [*Katz et al*., 1977; *Whipple*, 1981; *Jurac et al*., 1995]

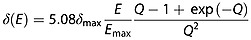
where *E* is the average energy of the incident electrons, *δ*_max_ is the maximum secondary emission yield, *E*_max_ is the energy at which the maximum yield occurs, and *Q* = 2.28(*E*/*E*_max_)^1.35^. We assume a predominantly water ice surface and use the values reported by *Jurac et al*. [1995] of *E*_max_ (340 eV) and *δ*_max_ (2.35) for water ice. The plasma parameters at Hyperion during the time of the encounter were based on a combination of estimated and measured values and are given in Table 1. The photoelectron current *I_p_* was taken from that of *Sternovsky et al*. [2008] at the subsolar point of the Moon during solar minimum and scaled to the orbital distance of Saturn (9.09 AU). The photoelectron and secondary electron distributions were assumed to be Maxwellian, with temperatures of 2 eV and 3 eV, respectively, as given by *Jurac et al*. [1995].

In order to evaluate surface charging of the downstream (wakeside) hemisphere, we have used the equations for static plasma from *Manka* [1973] with the same plasma parameters as listed in Table 1. As the gyroradius of the thermal ions is ∼4000 km, over an order of magnitude greater than the radius of Hyperion, only a small fraction of the ions will be absorbed by the moon, while the remaining ions may gyrate into the wake, helping to smooth out any plasma density depletion. Similarly, during several close flybys of Saturn's moon Rhea, Cassini failed to detect any significant decrease in plasma density within the expected plasma wake [*Roussos et al*., 2012]. Thus, while our treatment of the downstream hemisphere does not fully capture charged particle dynamics within the wake, it is a reasonable approximation.

**Figure 5:**
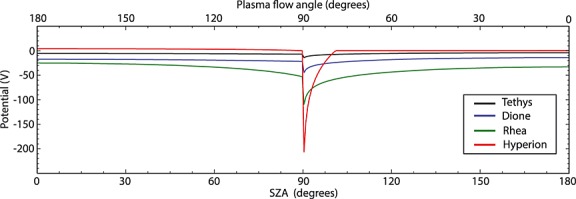
Predicted surface potential versus solar zenith angle (red) for Hyperion near dawn (0600) LT using the parameters described in Table 1. Predicted surface potentials of Tethys, Dione, and Rhea for the same configuration are shown for comparison.

Shown in Figure 5 is the expected surface potential profile for Hyperion given the ambient plasma conditions listed in Table 1. We show the expected potential profiles for Tethys, Dione, and Rhea calculated with the typical plasma parameters of *Roussos et al*. [2010] for comparison. We note that at the same configuration (i.e., near 0600 LT), the surface potential of Hyperion is expected to be less negative than those of the major moons in the inner magnetosphere, with a potential of near ∼0 V at most solar zenith angles. On the upstream hemisphere facing the plasma flow, this is due to the fact that at the high ambient electron temperature at Hyperion the secondary emission yield (*δ*) approaches unity, and thus, the small positive current from the ion flow term acts to suppress negative charging, even in shadow. However, as we go toward smaller solar zenith angles (SZA < 95°), the ion flow current is reduced, and we observe a shift toward strongly negative potentials that exceed −200 V near the solar terminator (SZA = 90°). On the downstream hemisphere, the photoemission current dominates over the contribution from the plasma currents and the surface reaches a slightly positive potential. Due to the fact that the plasma density at Hyperion is very low, even considerable changes to the plasma and electron temperature in the wake would not lead to any significant changes to the expected surface potentials on this hemisphere. Recent in situ measurements of secondary emission yields from the lunar surface have shown that the effective yield is a factor of ∼3 lower than what was expected from previous laboratory studies, possibly due to surface roughness effects [*Halekas et al*., 2009a]. If a similar reduction in the secondary emission yield would be applicable to Hyperion, we would expect the upstream hemisphere to reach large negative potentials due to the contribution from the plasma electron current, while the potentials at the sunlit downstream hemisphere would generally remain slightly positive due to the dominance of the photoemission current.

## 4 Discussion and Conclusions

Surface charging at Earth's Moon has been extensively investigated through spacecraft observations [*Halekas et al*., 2002, 2005, 2007, 2008, 2009a, 2009b] and through theoretical studies [*Manka*, 1973; *Farrell et al*., 2007; *Stubbs et al*., 2007, 2014; *Poppe and Horányi*, 2010; *Poppe et al*., 2012]. These studies have generally found that the dayside lunar surface is charged a few volts positive (∼10 V) and that the lunar nightside and terminator regions reach strongly negative potentials (∼ −100 to −200 V). On the lunar nightside, extreme charging (∼ −4.5 keV) has been found to occur when the Moon is outside of the Earth's magnetosphere and exposed to solar energetic particle events [*Halekas et al*., 2009b]. During times when the Moon is in the terrestrial plasma sheet, negative surface potentials of up to −2 kV have been observed, intriguingly above both sunlit and shadowed surfaces [*Halekas et al*., 2005]. By observing electron pitch angle distributions using the Electron Reflectometer instrument on board the Lunar Prospector spacecraft, *Halekas et al*. [2002] found evidence of field-aligned upward going electron beams originating from the lunar night side. These were explained as being due to secondary electrons emitted at low energies and subsequently accelerated by an electrostatic potential at the surface, with a central energy proportional to the potential difference between the spacecraft and the lunar surface.

Based on the above observations, we interpret that we have remotely detected a strongly negative surface potential on Hyperion, through the detection of electrons, likely secondary or photoelectrons, which have been accelerated by a significant electrostatic potential at the surface. The inferred surface potential is proportional to the potential difference between the spacecraft and the moon's surface and is on the order of −200 V based on the energy of the field-aligned electron populations observed by CAPS-ELS.

This is consistent with the fact that several instruments (MAG, CAPS-ELS, and MIMI-LEMMS) indicate that the spacecraft is likely to be magnetically connected to the surface of Hyperion. In addition, observations made by the RPWS instrument indicate the presence of electrostatic wave activity that is consistent with the presence of an electron beam. The combination of the midenergy electron flux enhancement observed by CAPS-ELS, absorption at higher energies in both CAPS-ELS and MIMI-LEMMS data, the wave activity observed by RPWS, all near closest approach and likely during a magnetic connection to the moon very strongly suggest that this multitude of features were moon-related.

Using estimated plasma parameters for Hyperion during the encounter, we have investigated the predicted surface potentials and found that we expect strongly negative potentials, on the order of −200 V, to be present near the solar terminator. We note that our calculations assume a perfectly spherical body and do not take into account local shadowing due to topography. As both the topography and overall shape of Hyperion is highly irregular, it is likely that a significant amount of local shadowing will occur, particularly inside deep craters. Such local shadowing effects have previously been investigated for the Earth's Moon [e.g., *Farrell et al*., 2007; *Poppe et al*., 2012], and it has been suggested that topography (e.g., craters and mountains) may produce locally enhanced electric fields due to shadowing from plasma and solar UV [*Farrell et al*., 2007]. As such, an estimate of the expected surface charging profile at Hyperion purely as a function of incident plasma flow and solar zenith angles may not be entirely accurate without also taking into account a more detailed shape model of the surface.

While we do not know the exact location of the surface footprint of the magnetic connection between the Cassini spacecraft and Hyperion, we note that the inferred potential is of the order of what is theoretically predicted for the solar terminator region. However, it is plausible that such potentials may also occur elsewhere on the surface due to Hyperion's irregular shape and rugged topography.
